# The Role of Traditional Plant Knowledge in the Fight Against Infectious Diseases: A Meta-Analytic Study in the Catalan Linguistic Area

**DOI:** 10.3389/fphar.2021.744616

**Published:** 2021-10-11

**Authors:** Airy Gras, Montse Parada, Joan Vallès, Teresa Garnatje

**Affiliations:** ^1^ Institut Botànic de Barcelona (IBB, CSIC-Ajuntament de Barcelona), Barcelona, Spain; ^2^ Laboratori de Botànica—Unitat Associada CSIC, Facultat de Farmàcia i Ciències de l’Alimentació—Institut de Recerca de la Biodiversitat IRBio, Universitat de Barcelona, Barcelona, Spain; ^3^ Secció de Ciències Biològiques, Institut d’Estudis Catalans, Barcelona, Spain

**Keywords:** Balearic islands, Catalan linguistic area, Iberian Peninsula, infectious diseases, multidrug resistance, plant medicinal uses, traditional knowledge

## Abstract

Infectious diseases represent, as a group, the main cause of mortality in the world. The most important reasons are multidrug-resistant pathogens, the rapid spread of emerging diseases aggravated by globalization, and the extended reach of tropical and vector-borne diseases resulting from continued climate change. Given the increase in these diseases and the limited effectiveness of antibiotics, traditional knowledge can constitute a useful tool to address these new health challenges. The aim of this work is to analyze extensively the available ethnobotanical data linked to infections and infestations in the Catalan linguistic area, with the intention of depicting the panorama of the folk use of herbal products to address the quoted ailments, preserving the popular plant knowledge and management data. The meta-analytic work performed in the present study covers 29 research studies belonging to different regions throughout the Catalan linguistic area. The medicinal ethnoflora to treat infections and infestations in the Catalan linguistic area includes 326 taxa belonging to 78 botanical families of vascular plants. The informant consensus factor (F_IC_) was 0.92, and the ethnobotanicity index (EI) was 7.26%. *Artemisia absinthium* (10.98%; 0.37) and *Thymus vulgaris* (8.06%; 0.27) are the most quoted taxa and have the highest values of the cultural importance index. The most reported use was antihelminthic (30.15%), followed by internal antiseptic (19.43%) and antipyretic (13.69%). The medicinal importance index shows the relevance of the antihelminthic use (14.23) and also the use against measles (10.19). The information is coincidental with at least one of the comprehensive pharmacological literature sources checked for 47.42% of ethnobotanical uses. These results, centered on the plants used to treat infection and infestation diseases, are the first step toward selecting some of the most interesting species to develop phytochemical and pharmacological studies and suggesting an alternative regarding how to face the health emergency involving the expansion of infectious diseases, based on local and traditional knowledge.

## Introduction

Infectious diseases represent, as a group, the main cause of mortality in the world. Annually, 15% of all deaths worldwide are directly attributable to infectious diseases (WHO (World Health Organization), 2018). The most important reasons are multidrug-resistant pathogens, the rapid spread of emerging diseases aggravated by globalization, and the extended reach of tropical and vector-borne diseases resulting from continued climate change (Eckhardt et al., 2020). All the aforementioned reasons are directly or indirectly due to mismanagement by humans.

One of the biggest challenges of medicine is the growth of multidrug-resistant microorganisms endangering the effectiveness of common drugs used in the health system (WHO (World Health Organization), 2015). Although multidrug resistance is a natural phenomenon, the inappropriate use of antimicrobial drugs, inadequate sanitary conditions, improper food handling, and poor infection prevention and control practices contribute to the emergence and promote the spread of resistance to several drugs (Tanwar et al., 2014). Without an effective action to reverse current trends, we could face a return to the pre-antibiotic era, with simple wounds and infections causing significant harm and even death, and routine medical procedures becoming a very high risk. It has been estimated that antimicrobial resistance (AMR) might cause more deaths than cancer by 2050 (EC (European Comission), 2017).

In addition, globalization accelerates the dispersion of microorganisms. Microorganisms, including viruses, move constantly, transported by humans, animals, plants, or move freely through the environment, and nowadays, they do so on a global scale driven by the international transport of merchandise and products, ballast water, and the exponential growth of human travel by air, sea, and land (Zhu et al., 2020). A clear example of this widespread movement is the SARS-CoV-2, a virus causing COVID-19, whose pandemic is affecting 195 countries and territories, with around 187,755,275 infected subjects and 4,048,124 deaths (CSSE (Center for Systems Science and Engineering), 2021).

In the same way, climatic factors promote the emergence and re-emergence of infectious diseases. The geographic expansion of microbes and vectors into new territories is favored by the sustained increase in temperature at higher latitudes, exposing all planetary lives to climate-sensitive infections, some of them previously unknown in particular habitats (Coates and Nortan, 2021).

Different pathogens, such as virus, bacteria, or protozoans, have been and continue to be the cause of major pandemics and epidemics around the world, some considered relatively mild, such as influenza, and some others qualified as severe, such as the aforementioned COVID-19. Some examples are *Vibrio cholerae*, cholera-causing bacteria; *Aedes aegypti*, the dengue-transmitting mosquito; or *Morbillivirus*, a genus of paramyxoviruses which causes measles.

Given the increase in these diseases and the limited effectiveness of antibiotics, traditional knowledge can constitute a useful tool to address these new health challenges. One of the most important recent drug designs from ethnobotanically used plants, having deserved a Nobel Prize in Physiology or Medicine, is the discovery of artemisinin (Tu, 2011), which began as a response to the resistance developed by plasmodia to quinine-derived drugs.

Local and traditional knowledge about medicinal plants, one of the objectives of the study of ethnobotany, is an intangible cultural heritage and a biodiversity resource in regression. Ethnobotany is, since its first description (Harshberger, 1896), a multifaceted discipline that studies plant names and their uses and management by human societies from ancient to current times, aiming at their projection to the present and future well-being of human societies (Pardo-de-Santayana and Macía, 2015). The majority of ethnobotanical work has focused on ethnopharmacology, with the possibility of using some of the collected information in the drug development (Heinrich and Jäger, 2015). Biodiversity, including traditional knowledge, plays an important role as a tool to support the fight against AMR and to solve the health emergency linked to this problem. In this way, the study of traditional knowledge can contribute to the One Health approach (WHO (World Health Organization), 2017), especially to the multidrug resistance of microorganisms.

The Catalan linguistic area (CLA) is one of the most largely studied territories in Europe from the ethnobotanical point of view (Vallès, 2019). In particular, the Catalan ethnoflora was also studied in terms of life forms and distribution, and the results show the importance of using plants with a large distribution area and plants with available biomass throughout the year (Gras et al., 2020a). The extensive information on plants used to treat infections and infestations emerges in the recently published meta-analytic study about the role of botanical families in medicinal ethnobotany of this area (Gras et al., 2021). This kind of traditional medicinal use has been shown to be relevant in different geographic and cultural areas (Phumthum and Balsev, 2020, and references therein).

In this context, the aim of this work is to analyze extensively the available ethnobotanical data linked to infections and infestations in CLA, with the intention of depicting the panorama of the folk use of herbal products to address the quoted ailments, preserving the popular plant knowledge and management data. In addition, a phytotherapeutic and pharmacological literature comparison has been carried out, in order to provide a basis for further research and help in the development of medicinal products and to face the challenges of the future.

## Material and Methods

### Study Area

**FIGURE 1:**
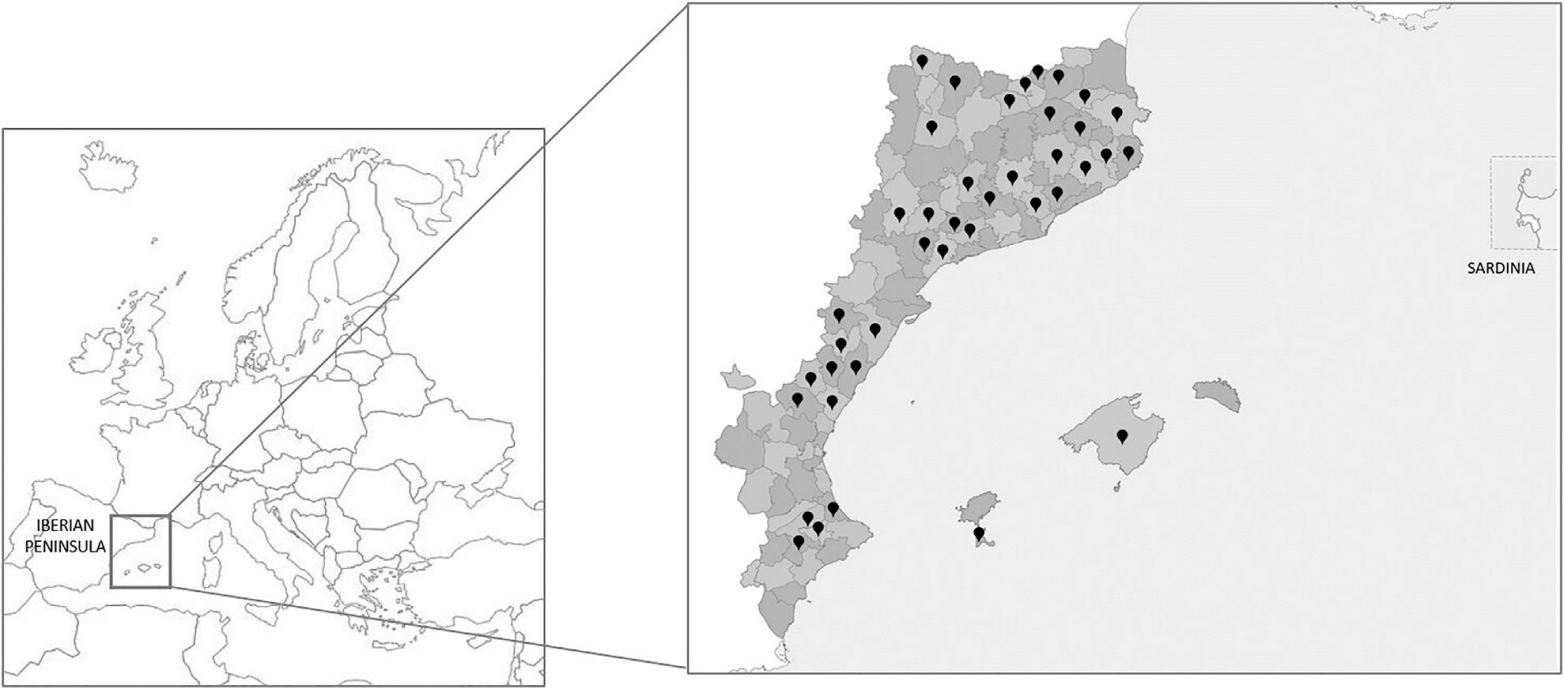
Map of the territories studied within Europe and the Catalan linguistic area. Dots indicate the areas with prospections analyzed in the present study.

The Catalan-speaking area, Catalan language territories, Catalan countries, or Catalan linguistic area constitute a well-studied unit with different approaches: geographic (Deffontaines, 1978), physiographic (Riba et al., 1984), floristic (Bolòs and Vigo, 1984; Bolòs et al., 2005), vegetation (Folch et al., 1984), and linguistic and cultural (Badia, 1966). This unit, located in the eastern part of the Iberian Peninsula, also includes a northern Pyrenean portion, the Balearic Islands and the city of L’Alguer on the island of Sardinia (Figure 1). Politically, these territories, with an extension of 70,000 km^2^ (Bolòs et al., 2005) and around 14,100,000 inhabitants (Departament d’Estadística del Govern d’Andorra, 2021; IBESTAT (Institut d’Estadística de les Illes Balears), 2021; IDESCAT (Institut d’Estadística de Catalunya), 2021; ISTAT (Istituto Nazionale di Statistica), 2021; Portal Estadístic de la Generalitat Valenciana, 2021), belong to four states: Andorra (all the territory), France (Northern Catalonia or eastern Pyrenees department), Italy (L’Alguer, Sardinia), and Spain (Balearic Islands, Carxe—a small area in Murcia, Catalonia, a portion of eastern Aragon, and Valencia).

From the Mediterranean Sea level to 3,143 m a.s.l. in Pica d’Estats (Pyrenees), the landscape of the area considered is structured in several stages with distinct floristic and vegetation traits (Folch et al., 1984; Bolòs et al., 2005), harboring approximately 4,300 autochthonous and 1,200 allochthonous (Sáez, 2019)^1^ plant taxa, including species and subspecies.

### Databasing and Data Selection

The information has been collected through semi-structured ethnobotanical interviews (Gras et al., 2020b and references therein) following the ethical principles of the International Society of Ethnobiology (ISE (International Society of Ethnobiology), 2021) and included in a webpage (https://etnobotanica.iec.cat). The herbarium vouchers are deposited in the herbarium BCN (Centre de Documentació de Biodiversitat Vegetal, Universitat de Barcelona). The meta-analytic work carried out in the present study covers 29 research studies, performed between 1990 and 2019, belonging to different regions throughout the CLA, and available in the open access webpage mentioned before.

The information concerning infections and infestations has been recovered from the mentioned database. A minor bias exists because in one out of the 29 studies included in the dataset (Mulet, 1990), each taxon is assigned to a municipality, instead of an informant, as is the case in all the other works. This can result in a slight underestimation of used reports and also of the indexes that include them. Neither fungi nor non-vascular plants are considered. For taxa nomenclature, Bolòs et al.’s (2005) study has been followed, which is a flora covering specifically the area considered, and for family attribution we follow APG IV, the last Angiosperm Phylogeny Group’s arrangement to date (APG (The Angiosperm Phylogeny Group), 2016).

### Data Analyses

All descriptive statistics and quantitative ethnobotany were carried out using Excel (Microsoft Excel 2016). To analyze the results, we have used the use report (hereinafter, UR) (Vandebroek et al., 2008). In addition, with the aim of assessing the state of knowledge, some ethnobotanical indices were applied: 1) the ethnobotanicity index (EI; Portères, 1970), the quotient between the number of plant taxa used (here taking into account the plants used for infectious diseases) (n), and the total number of plant taxa that constitute the flora of the territory (autochthonous plants, see an earlier estimation) (N), expressed as a percentage, in order to have a general idea of the relevance of these plants in the area considered; [EI=(n/N)*100] 2) informant consensus factor (F_IC_; Trotter and Logan, 1986), the ratio of the number of UR minus the number of plant taxa used (n) to the number of UR minus one, in order to assess the consistency or robustness of the traditional knowledge regarding infection and infestation in the territory [F_IC_= (UR–n)/(UR–1)]; 3) the cultural importance index (CI; Tardío and Pardo–De-Santayana, 2008), the sum of the proportion of informants that mention each taxon use, has also been calculated to identify the plants most valued by the informants [CI = Σ^uNC^_u=u1_ Σ^iN^_i=i1_ URui/n]; and 4) the medicinal importance index (MI; Carrió and Vallès, 2012), the quotient between the total UR for a specific use category and the number of plant taxa possessing this use (n), to evaluate the real importance of the use [MI=UR/n].

### Pharmacological Comparison in the Literature

To compare, whenever possible, the activity of the plants reported in pharmacological sources, a revision of the literature is carried out for the most reported plants, those with three or more quotations for a particular use. The pharmacological comparison has been performed using monographs from official sources and encyclopedic bibliography on phytotherapy (ESCOP (European Scientific Cooperative on Phytotherapy), 2021; EMA (European Medicines Agency), 2021; Fitoterapia.net, 2021; Blumenthal, 2003; Duke, 2003).

## Results and Discussion

### Characteristics of the Interviewees

Data were gathered from 1,274 informants. Mainly, the informants were born in the studied area or have been living there a very significant part of their lives, and most of them are professionally related to agricultural and livestock-raising activities.

### General Data

The total number of plant taxa claimed to be useful to cure infections and infestations in the CLA, taking into account the dataset mentioned earlier, is 326. Six of the total taxa were determined only at a generic level and 33 at an infraspecific level. All these taxa are distributed among 78 families. The number of use reports collected for these taxa is 4,282. The complete dataset of the recorded plants used to treat infections and infestations in the studied area is available in the appendix: Supplementary Material S1. The numbers of taxa and families recorded are high. Just to quote an example from a different geographical and cultural zone in a botanically rich area, the numbers of plant taxa and botanical families by one of Thailand’s ethnic groups, the Karen, to fight against infectious troubles were 127 and 59, respectively (Phumthum and Balsev, 2020).

It is worth mentioning that in the studied area, plants are also traditionally used against infections and infestations in animals. The ethnoveterinary uses are much less numerous than those addressed to human medicine: 725 use reports of 112 taxa belonging to 49 families. These data will not be studied in the present work, but they could be relevant in a future global analysis of the ethnoveterinary knowledge in the CLA.

With a view to assessing the state of ethnobotanical knowledge about the plants used to treat infections and infestations in the CLA, the informant consensus factor (F_IC_) was 0.92 (the maximum value for this parameter is the unit). This value indicates a strong agreement among the informants for the plants used in the treatment of these illnesses in the studied area, accounting for the robustness and the consistency of the dataset. In some previous studies, the values obtained for this parameter were 0.83 for the treatment of respiratory tract infectious diseases (Rigat et al., 2013) and 0.93 for topical uses (Rigat et al., 2015), and the infection and infestation F_IC_ value demonstrated the high consistency of data presented here. In the first example, the consistency could be biased by the small number of informants in a very restricted area. The ethnobotanicity index (EI), calculated by only taking account the autochthonous taxa recorded (those included in Bolòs et al., 2005), is 7.26% for the studied area; this means that roughly one-tenth of the plants of the area have been claimed as useful by the informants. The EI in the territories considered mostly the ranges from 22 to 29%, and the results for uses against infections and infestations, with more than 7%, constitute a significant part, around one-quarter.

### Most Reported Taxa and Parts of Plant Used

Among the five most cited families, which represent more than half of total use reports (54.39%), we find some of the most relevant in ethnobotanical studies in the Mediterranean, namely, Asteraceae (17.80%), Lamiaceae (16.37%), but also Amaryllidaceae (6.05%), Caprifoliaceae (4.83%), Adoxaceae (4.67%), and Rutaceae (4.67%). On the one hand, the high percentages raised by Asteraceae and Lamiaceae are not surprising because of their abundance in absolute terms and in the Mediterranean flora. On the other hand, the remaining families are not among the most cited in other similar studies, which makes them especially interesting as potentially useful in the treatment of the pathologies presented here. Even if the antibacterial properties of some species such as garlic, belonging to the Amaryllidaceae family, are well-known, further studies on other species in the same family might be advisable, especially now that after the last restructuring of orders and families (APG (The Angiosperm Phylogeny Group), 2016), they have ceased to be part of the Liliaceae family. Caprifoliaceae is not a commonly quoted family in ethnobotanical studies; the use of species of *Scabiosa* (Caprifoliaceae) to combat measles, one of the few uses of this genus, has been profusely cited in the past, but this use has been lost due to the existence of a vaccine against measles. Recently, *Sambucus nigra* L. has moved to Adoxaceae from the Caprifoliaceae family, and this species is one of the most cited across the CLA as an antiseptic, a fact that explains the position of this family among the most reported ones.

The most used taxa to treat infections and infestations have been *Artemisia absinthium* L. (10.98%) and *Thymus vulgaris* L. (8.06%), followed by *Allium sativum* L. (4.62%), *Sambucus nigra* (4.55%), and *Ruta chalepensis* L. (3.29%). The two most quoted species are well-known by the informants for their specific uses, in the case of *Artemisia absinthium*, as an antihelminthic (10.72%), and *Thymus vulgaris*, as an internal antiseptic (5.93%). The Catalan common name of *Artemisia absinthium* “herba cuquera” (exactly the same as the English wormwood) expresses its medicinal properties. In addition to the regular and abundant use, many magic and religious beliefs and practices are associated with this leading anti-infectious plant in the area studied. *Scabiosa atropurpurea* L. and *S. columbaria* L. are among the five most quoted taxa (Table 1), which is uncommon in general medicinal ethnofloristic studies. The *Scabiosa* genus is widely used in the CLA to treat measles in tisane form. Traditionally, in some studied areas, this treatment was accompanied by other actions, such as covering the lights in the patient’s room with a red cloth (Agelet, 1999).

**TABLE 1:** 25 most quoted plants used for infection and infestation in the studied area, with the most quoted medicinal uses (≥3 UR) and the number of total use reports and percentage. Comparation of uses in pharmacological comprehensive literature: ^1^ EMA (European Medicines Agency), 2021, ^2^ ESCOP (European Scientific Cooperative on Phytotherapy), 2021, ^3^ Fitoterapia.net (2021), ^4^ Duke (2003).

Taxon (family)	Medicinal use (≥3 UR)	Total UR	Total UR (%)	CI index
*Artemisia absinthium* L. (Asteraceae) BCN 29837	Antihelminthic,^1,4^ antipyretic^4^, and for brucellosis	470	10.98	0.37
*Thymus vulgaris* L. (Lamiaceae) BCN 96764	Antihelminthic,^4^ for cold,^1,3,4^ for flu, for tonsillitis,^3^ for whitlow, and internal antiseptic^3,4^	345	8.06	0.27
*Allium sativum* L. (Amaryllidaceae) BCN 29832	Antibacterial,^3,4^ antihelminthic,^4^ antiherpetic,^3,4^ antipyretic,^4^ antityphoid^4^, and internal antiseptic^4^	198	4.62	0.16
*Sambucus nigra* L. (Adoxaceae) BCN 96771	Antiparotitis, antipneumonic,^4^ antipyretic,^4^ antityphoid, for cold,^1,2,3,4^ for brucellosis, for erysipelas,^4^ for flu,^3,4^ for tonsillitis, and internal antiseptic	195	4.55	0.15
*Ruta chalepensis* L. (Rutaceae) BCN 140153	Antihelminthic, for cold, and internal antiseptic	141	3.29	0.11
*Scabiosa atropurpurea* L. (Caprifoliaceae) BCN 125416	Antivariolous, and for measles	122	2.85	0.10
*Vitis vinifera* L. (Vitaceae) BCN 150353	Antifungal,^4^ antihelminthic, antiherpetic,^2,4^ antipneumonic, antipyretic,^4^ antityphoid, internal antiseptic,^4^ for cold, for lice infestations, and for tonsillitis	113	2.64	0.09
*Olea europaea* L. subsp. *europaea* var. *europaea* (Oleaceae) BCN 125505	Antifungal, antihelminthic, antitetanic, antityphoid, for cold, for tonsillitis, and internal antiseptic	92	2.15	0.07
*Carum carvi* L. (Apiaceae) BCN 24739	Internal antiseptic^4^	82	1.91	0.06
*Scabiosa columbaria* L. (Caprifoliaceae) BCN 24993	Antipyretic, and for measles	78	1.82	0.06
*Rosmarinus officinalis* L. (Lamiaceae) BCN 156603	Antipneumonic, for cold, for flu, and internal antiseptic	70	1.63	0.05
*Eucalyptus globulus* Labill. (Myrtaceae) BCN 29696	Antipneumonic, antipyretic, for cold^1,4^, and for flu	63	1.47	0.05
*Triticum aestivum* L. (Poaceae) BCN 156578	Antifungal, antihelminthic, antipneumonic, antipyretic,^4^ for cold, and for tonsillitis	61	1.42	0.05
*Allium cepa* L. (Amaryllidaceae) BCN 28655	Antihelminthic,^4^ antipneumonic, antityphoid, and for whitlow,^4^ and internal antiseptic^4^	59	1.38	0.05
*Brassica oleracea* L. subsp. *oleracea* (Brassicaceae) BCN 32181	Antipyretic, and internal antiseptic.	59	1.38	0.05
*Gentiana lutea* L. (Gentianaceae) BCN 29700	Antihelminthic^4^, and internal antiseptic^4^	55	1.28	0.04
*Gentiana burseri* Lap. subsp. *burseri* (Gentianaceae) BCN 83629	Antihelminthic, and internal antiseptic	50	1.17	0.04
*Malva sylvestris* L. (Malvaceae) BCN 125508	Antipyretic,^4^ for cold,^4^ and internal antiseptic^4^	50	1.17	0.04
*Santolina chamaecyparissus* L. (Asteraceae) BCN 96763	Antihelminthic,^3,4^ antipyretic, for flu, and internal antiseptic^3^	45	1.05	0.04
*Juniperus communis* L. (Cupressaceae) BCN 29878	Antifungal,^3,4^ antihelminthic,^4^ for flu,^4^and internal antiseptic^4^	42	0.98	0.03
*Lavandula angustifolia* Mill. subsp. *pyrenaica* (DC.) Guinea (Lamiaceae) BCN 29881	Antihelminthic^4^, and internal antiseptic^4^_._	39	0.91	0.03
*Mentha spicata* L. (Lamiaceae) BCN 125414	Antihelminthic^4^, and internal antiseptic^4^	38	0.89	0.03
*Tanacetum parthenium* (L.) Sch. Bip. (Asteraceae) BCN 29960	Antipyretic^4^, and internal antiseptic^4^	36	0.84	0.03
*Citrus limon* (L.) Burm. (Rutaceae) BCN 150354	Antihelminthic, for cold,^4^ and internal antiseptic^3^	35	0.82	0.03
*Pinus halepensis* Mill. (Pinaceae) BCN 150381	Antipneumonic, for cold, and for tonsillitis	34	0.79	0.03

Out of the 326 specific and infraspecific taxa recorded, 183 (56.13%) fit the reliability requisites indicated by Le Grand and Wondergem (1987) and Johns et al. (1990) of having three or more reports from independent informants. This indicates a high degree of reliability of the information of this significant part of the dataset. Additionally, reports from less than three informants, apart from having an ethnographic and cultural interest, cannot be underestimated in terms of further research, since they could constitute the remnants of a more solid old system of traditional knowledge, now weaker due to acculturation or transculturation processes suffered by developed societies.

The cultural importance (CI) index is another indication of the appreciation that the informants have for the plants they use. Its values (Table 1) are the highest in the first plants of the UR ranking, the maximum being 0.37 for *Artemisia absinthium*. Combining these two indicators of taxa’s reliable use and appreciation could be of great value for selecting plants that would be of interest for deeper studies in the frame of drug development processes.

### Plant Parts Employed, Medicinal Uses, and Pharmaceutical Forms

The plant parts most commonly used to treat infections and infestations are the aerial part with 40.28%, including young, sterile, flowering, and fructified aerial parts; these organs are followed by flowers or inflorescences (16.79%) and fruits, infructescences, or fructifications (10.70%). This is not surprising since all the quoted plant parts are among those more apparent and so easily available.

In the studied area (CLA), the medicinal uses quoted are very diverse, and the infections and infestations addressed are caused by different pathogens. The principal groups of pathogenic organisms and the caused diseases are summarized in Supplementary Table S2. For a large number of diseases, the cause cannot be associated with a specific pathogen, and for this reason, many of them have been grouped in the category termed “various” (49.09%). Following this miscellaneous group, diseases provoked by helminths (30.22%) and viruses (10.46%) are the most important. Uses against bacteria (6.87%) and fungi (2.50%) have also been rather widely reported. Finally, the reports addressed to treat infections and infestations produced by insects (0.58%), arachnids (0.23%), or protozoans (0.05%) are minimal, taking into account that the insect repellents (183 UR) are not included in this analysis because they could be, in any case, preventive but not linked to infectious or infestation processes.

**FIGURE 2:**
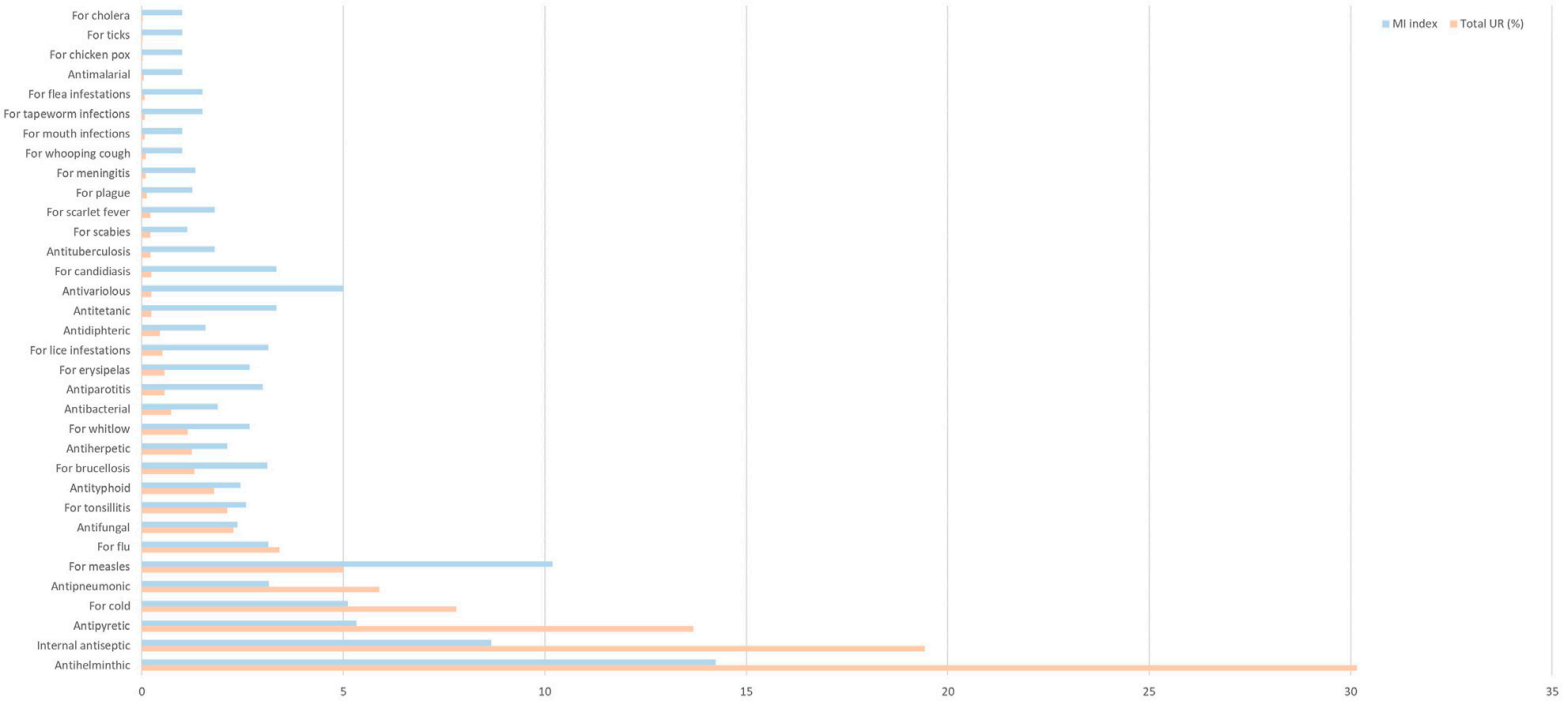
Total use report percentage of medicinal uses to treat infections and infestations, and their medicinal importance (MI) index.

Among the most quoted uses, we find the antihelminthic (30.15%), followed, although quite far, by the internal antiseptic (19.43%) and antipyretic (13.69%), both of them grouped in the category named “various pathogens,” since they are properties useful for a wide range of infections or infestations, irrespective of the causing agent. Some of the less frequent diseases, such as tuberculosis or meningitis, not common today, and diseases such as cholera or malaria, which can be qualified as exotic in the area considered, are quoted as well, but not prominently (Figure 2; Table 2).

**TABLE 2:** Medicinal uses to treat infections and infestations and values of total use reports, total use reports percentage and medicinal importance (MI) index in the studied territories.

Medicinal use	Total UR	Total UR (%)	MI index
Antihelminthic	1,291	30.15	14.23
Internal antiseptic	832	19.43	8.67
Antipyretic	586	13.69	5.33
For cold	334	7.80	5.11
Antipneumonic	252	5.89	3.15
For measles	214	5.00	10.19
For flu	146	3.41	3.13
Antifungal	97	2.27	2.37
For tonsillitis	91	2.13	2.59
Antityphoid	77	1.80	2.45
For brucellosis	56	1.31	3.11
Antiherpetic	53	1.24	2.12
For whitlow	49	1.14	2.67
Antibacterial	31	0.72	1.88
Antiparotitis	24	0.56	3.00
For erysipelas	24	0.56	2.67
For lice infestations	22	0.51	3.14
Antidiphteric	19	0.44	1.58
Antitetanic	10	0.23	3.33
Antivariolous	10	0.23	5.00
For candidiasis	10	0.23	3.33
Antituberculosis	9	0.21	1.80
For scabies	9	0.21	1.13
For scarlet fever	9	0.21	1.80
For plague	5	0.12	1.25
For meningitis	4	0.09	1.33
For whooping cough	4	0.09	1.00
For mouth infections	3	0.07	1.00
For tapeworm infections	3	0.07	1.50
For flea infestations	3	0.07	1.50
Antimalarial	2	0.05	1.00
For chicken pox	1	0.02	1.00
For ticks	1	0.02	1.00
For cholera	1	0.02	1.00

The index of medicinal importance is useful to evaluate the real importance of each use, the importance of the use category depending not only on the total number of UR but on the number of UR per taxon as well. This index was calculated for all the medicinal use categories, and the results range from 1 to 14.23 (Figure 2; Table 2). The highest medicinal importance index corresponds to the most quoted use, antihelminthic (14.23), followed by the use against measles (10.19). These high values show that these use categories have a low diversity of taxa in relation to the number of use reports.

Regarding the pharmaceutical forms, among the 41 employed, tisane, including decoction and infusion, represents 49.25% of the total forms reported, followed by direct use (internal or external) (13.73%) and poultice (10.63%). Roughly half the remedies are applied with a tisane, the most universal pharmaceutical form in ethnobotany, the direct ingestion or external use of the plant part without any preparation being also common in traditional knowledge, as well as poultices for external use (Gras et al., 2020b).

### Pharmacological Comparison

The top 25 taxa and their main medicinal uses with three or more use reports (Table 1) were compared with a literature set constituted by some comprehensive or encyclopedic works with plant pharmacological data (EMA (European Medicines Agency), 2021; ESCOP (European Scientific Cooperative on Phytotherapy), 2021; Fitoterapia.net (2021); Blumenthal, 2003; Duke, 2003). The information was coincidental with at least one of the sources checked for 47.42% of ethnobotanical uses. By far, Duke’s CRC Handbook of Medicinal Herbs (2003) with 44.33% of coincidental uses and Fitoterapia.net (2021) with 11.34% were the most inclusive, systematic and detailed works analyzed. The traditional uses confirmed in this literature set, which is often used in drug registration processes, may be considered as the most consolidated. In addition, roughly 53% of the ethnobotanical uses (all of them reliable in terms of the number of three or more use reports Le Grand and Wondergem, 1987; Johns et al., 1990) not found in the literature set should be the object of further phytochemical and/or pharmacological investigation.

## Concluding Remarks

The medicinal ethnoflora to treat infections and infestations is wide and well-known in CLA, with a total of 326 taxa used. The values of the informant consensus factor (F_IC_) and the ethnobotanicity index (EI) also indicate the robustness of this traditional knowledge in the studied area.

The information was compared with at least one of the comprehensive pharmacological literature sources checked for 47.42% of ethnobotanical uses. These plants and uses will be the most likely candidates for drug development programs, and at the same time, a need of further phytochemical and medicinal activity studies for those traditional uses not found in the pharmacological literature will arise.

These results centered on the plants used to treat infection and infestation are the first step toward selecting some of the most interesting species to develop phytochemical studies and serve to suggest an alternative as to how to face the health emergency concerning the expansion of infectious diseases and the drug resistance of many pathogens, based on local and traditional knowledge. Once again, ethnobotany reveals itself as a relevant first step in drug design and development processes and plays an important role in new crises such as the AMR.

## Data Availability

The original contributions presented in the study are included in the article/Supplementary Material; further inquiries can be directed to the corresponding author.

## Footnotes

1 Sáez, L. (2019). Bellaterra, Catalonia, Spain: Universitat Autònoma de Barcelona. personal communication
