# Supplementary material for: The Role of Traditional Plant Knowledge in the Fight Against Infectious Diseases: A Meta-Analytic Study in the Catalan Linguistic Area
Source: Front Pharmacol. 2021 Oct 11;12:744616. doi: 10.3389/fphar.2021.744616 (PMC8543157; doi:10.3389/fphar.2021.744616)
Supplement: Supplementary file 1 [file Table2.docx]

**Supplementary material 2.** Pathogens and diseases caused

| **Group of pathogens** | **Pathogen** | **Diseases** |
| --- | --- | --- |
| Arachnids | *Ixodes ricinus* | Tick infestation |
|  | *Sarcoptes scabiei* | Scabies |
| Bacteria | *Bordetella pertussis* | Whooping cough |
|  | *Brucella melitensis*, *B. abortus* and *B. Suis* | Brucellosis |
|  | *Clostridium tetani* | Tetanus |
|  | *Corynebacterium diphteriae* | Diphteria |
|  | *Staphylococcus* and *Streptococcus* genera | Whitlow |
|  | *Mycobacterium tuberculosis* | Tuberculosis |
|  | *Salmonella typhi* | Typhoid |
|  | *Streptococcus* β-hemolytic | Scarlet fever |
|  | *Streptococcus* group A | Erysipelas |
|  | Various bacteria | Bacterial infections |
|  | *Vibrio cholera* | Cholera |
|  | *Yersinia pestis* | Plague |
| Fungi | *Candida albicans* | Candidiasis |
|  | Various fungi | Fungal infections |
| Helminths | *Taenia*, *Echinococcus*, *Dipylidum* and *Hymenolepis* genera | Tapeworm infections |
|  | Various helmiths | Helminthiasis |
| Insects | *Pediculus humanus capitis* | Lice infestations |
|  | *Pulex irritans*, *Xenopsylla cheopis* | Tapeworm infections |
| Protozoa | *Plasmodium* genus | Malarial |
| Various | _ | Fever |
|  | _ | Pneumonia |
|  | _ | Sepsis processes |
|  | _ | Mouth infections |
|  | _ | Meningitis |
|  | _ | Cold |
|  | *Streptococcus* β-hemolytic A and various viruses | Tonsillitis |
| Viruses | *Herpesviridae* family | Herpes |
|  | *Human herpesvirus* 3 | Chicken pox |
|  | Myxoviruses | Flu |
|  | *Morbillivirus* genus | Measles |
|  | *Paramyxovirus* genus | Parotiditis |
|  | *Poxvirus variolae* | Variole |
